# Clients’ perceptions of barriers and facilitators to implementing hepatitis C virus care in homeless shelters

**DOI:** 10.1186/s12879-020-05103-6

**Published:** 2020-05-29

**Authors:** Carmen L. Masson, J. Konadu Fokuo, August Anderson, Jesse Powell, Barry Zevin, Dylan Bush, Mandana Khalili

**Affiliations:** 1 Department of Psychiatry, University of California San Francisco, Zuckerberg San Francisco General, Hospital and Trauma Center, 1001 Potrero Avenue, Building 20, Suite 2100, San Francisco, CA 94110 USA; 2 Department of Medicine, University of California San Francisco, Zuckerberg San Francisco General, Hospital and Trauma Center, 1001 Potrero Avenue, NH-3D, San Francisco, CA 94110 USA; 3 Hennepin Healthcare, 715 South 8th Street, Minneapolis, MN 55404 USA; 4 Street Medicine and Shelter Health, at the San Francisco Department of Public Health, 50 Ivy St, San Francisco, CA 94102 USA; 5 Department of Medicine, University of California San Francisco, Zuckerberg San Francisco General, 1001 Potrero Avenue, NH-3D, San Francisco, CA 94110 USA

**Keywords:** Focus group, Homeless, Drug use, Mental illness, HCV testing, DAA treatment

## Abstract

**Background:**

Hepatitis C virus (HCV) is highly prevalent among homeless persons, yet barriers continue to impede HCV testing and treatment in this population. We studied the experiences of homeless individuals related to accessing HCV care to inform the design of a shelter-based HCV prevention and treatment program.

**Methods:**

Homeless shelter clients (10 women and 10 men) of a large shelter in San Francisco participated in gender segregated focus groups. Focus groups followed a semi-structured interview format, which assessed individual, program/system, and societal-level barriers and facilitators to universal HCV testing and linkage to HCV care. Focus group interviews were transcribed, coded, and analyzed using thematic analysis.

**Results:**

We identified key barriers to HCV testing and treatment at the individual level (limited knowledge and misconceptions about HCV infection, mistrust of health care providers, co-morbid conditions of substance use, psychiatric and chronic medical conditions), system level (limited advocacy for HCV services by shelter staff), and social level (stigma of homelessness). Individual, system, and social facilitators to HCV care described by participants included internal motivation, financial incentives, prior experiences with rapid HCV testing, and availability of affordable direct acting antiviral (DAA) treatment, respectively.

**Conclusions:**

Interrelated individual- and social-level factors were the predominant barriers affecting homeless persons’ decisions to engage in HCV prevention and treatment. Integrated models of care for homeless persons at risk for or living with HCV address many of these factors, and should include interventions to improve patient knowledge of HCV and the availability of effective treatments.

## Background

Persons who are homeless and marginally housed have higher rates of serologic evidence of past or current hepatitis C virus (HCV) infection as compared to an estimated prevalence of 1.7% for all U.S. adults [1]. As compared with the general population, higher rates of HCV prevalence have been documented among community samples of homeless and marginally housed people in San Francisco at 46% [2], and in the Skid Row of Los Angeles at 86% [3]. Injection drug use is the primary route of HCV transmission in the general population [4], and similarly an independent risk factor for HCV infection among homeless populations [2, 5, 6]. Other risk factors associated with HCV infection among homeless adults include non-injection illicit drug use [5], history of incarceration [5, 6], and mental illness [2]. These overlapping risk factors not only increase a homeless person’s risk for HCV, but are also associated with poor access to health care and complicate the delivery of care for this population [6].

Although the treatment of HCV infection with new direct acting antiviral (DAA) medications results in high cure rates following completion of treatment, gaps in the HCV treatment cascade persist [7]. In the U.S., most people infected with HCV are uninsured or are insured by government-sponsored programs (i.e., Medicare and Medicaid programs) [8]. Due to the high cost of the DAAs, some state Medicaid programs impose restrictions on access to HCV treatment based on strict alcohol and drug utilization criteria contributing to disparities in access to HCV treatment [9, 10]. Studies conducted after the introduction of DAAs show wide variation in HCV treatment initiation rates following referral depending on the treatment setting. For example, in a study evaluating the HCV care continuum among patients receiving care at an urban network of five federally qualified health centers (FQHC), only 15% initiated treatment [11]. Similarly, low rates of HCV treatment initiation were found among formerly incarcerated individuals receiving care in a FQHC, with only 10% initiating treatment [12]. Among chronic HCV patients receiving care at four large urban hospital systems, the overall treatment rate was 17% [10].

Recent studies examining predictors of DAA treatment uptake suggest a lower likelihood of DAA treatment initiation among people who are racial/ethnic minorities [13, 14], have a substance use problem [13, 15], have government sponsored insurance [10, 13], and have issues with insurance or medication access [14]. Other common reasons for low treatment uptake include a lack of follow up [14, 15] and failure to obtain laboratory testing [15]. These factors are especially prevalent in the homeless population, but data on barriers to HCV care and treatment uptake in the DAA era among people who are homeless is limited. In one study of DAA initiation rates for homeless-experienced individuals in a patient centered medical home model of primary care, only 59% initiated treatment following referral [16].

HCV education, point-of-care testing, and treatment can be offered in homeless shelters. However, to develop effective programs tailored to address the complex health care needs of homeless populations, it is necessary to identify potential implementation barriers. Using focus groups of individuals accessing homeless shelters, this study contributes to the understanding of the barriers and facilitators to HCV care among homeless persons in the era of the DAAs to enable effective implementation of a universal HCV rapid testing and linkage to care model in homeless shelters.

## Methods

### Setting

This study was conducted in a large homeless shelter in San Francisco, which provides services to over 300 people per day. The shelter provided supportive housing, meals and a variety of services, including intensive case management, behavioral health services, and medical care. The shelter offered limited HCV testing and linkage to HCV services. Prior to recruitment, the research team worked with shelter management staff to design recruitment procedures and promotion strategies, and collaborated with shelter staff to implement the study.

### Procedures

A purposive sample of shelter clients was recruited to qualitatively describe experiences and perceptions related to accessing HCV care. Participants were recruited from shelter residents who were over 18 years of age using flyers posted and distributed by shelter staff. Participants were selected from sign-up lists of shelter clients who had indicated an interest to participate in one of two focus groups. Each sign-up sheet was limited to a maximum of 20 participants. Two separate focus groups for women and men were organized to increase comfort and reduce stigma associated with discussion of sensitive topics. The two focus groups provided sufficient data to respond to our research question with adequate depth [17]. Each 60-min focus group session was conducted between September and October 2018 at the participating shelter.

Focus groups interviews were conducted by a facilitator and co-facilitator who are clinical psychologists and have research expertise in the addictions field (CM and KF). The facilitators did not have any prior relationship with participants selected to participate in focus groups. The role of the co-facilitator was to take detailed notes, monitor the discussion, and debrief with the facilitator at the end of the group [18]. Prior to the start of the focus group, the facilitator reviewed the study’s purpose, the informed consent process, and discussed confidentiality. Following informed consent, participants completed a demographic survey. The facilitator used a semi-structured interview guide to conduct focus groups, which included open-ended questions such as, “What do you think are some reasons that people experiencing homelessness have for getting a hepatitis C test?” (Supplementary material). The facilitator used probes as needed to elicit more responses or expand ideas expressed by participants. The facilitator rephrased key statements back to participants during the interview process to verify accurate interpretation of participant responses [19]. Focus group discussions were digitally recorded and transcribed verbatim for thematic analysis. Participants were paid with a $25 gift card for participating in the focus group. The institutional review board of the University of California, San Francisco reviewed and approved study procedures.

### Qualitative data coding and analysis

Focus group interviews were checked for accuracy and de-identified. Transcribed focus groups were analyzed using qualitative thematic analysis [20]. We used the Health Behavior Framework (HBF) [21], a multidimensional model that synthesizes several models of health behavior, social theory, and change, to map factors that influence behavioral intentions, thereby influencing health behavior. The model considers barriers and supports at the individual (e.g., knowledge, health beliefs, cultural factors and beliefs), provider and health care system (e.g., practice patterns, health care setting), and societal level (e.g., impoverished neighborhoods) that may directly or indirectly influence health behaviors [21]. It is important to note that homelessness itself is socially determined, thus themes related to barriers to care could be grouped into more than one category [22]. Nonetheless, the use of this comprehensive model allows the simultaneous examination of the influence of behavioral intentions on health seeking behavior and the influence of the environment in which health care is received and enacted. We used the HBF to guide the analysis and interpretation of qualitative interview data.

In the first step of the data analysis, three readers (CM, KF, and AA) reviewed the transcripts to become familiar with the discussions that occurred in each focus group. In the second step, coders met as a group to conduct preliminary coding of the data to identify emerging themes from the text comprised of a line, sentence, or paragraph [23]. In the third step, coders assigned codes to the data and grouped them into categories and subcategories. Coders met on four separate occasions to compare coding choices, suggesting possible codes and definitions for the codebook. Once consensus was reached about the acceptability of code labels and definitions, coders used the refined codebook to independently assign codes to the transcripts. This iterative process of coding continued until all the text in the transcripts had been coded. The themes were then further analyzed to assess barriers and facilitators to HCV care at the individual, system, and societal levels. Illustrative quotations were chosen from the interviews.

## Results

### Study population characteristics

The sample included 10 women and 10 men with an age range of 26–69 years. Participants were predominantly racial/ethnic minorities (n = 15), while 5 identified as White. The majority of participants (n = 15) had completed a high school education or above. Eight reported a history of injection drug use; almost half (n = 9) had experienced more than one episode of homelessness, and 7 had been continuously living on the streets or in shelters without a place to stay for a year or longer. Of the 20 participants, 4 reported that they had tested positive for HCV. Among the 4 participants who disclosed their HCV status, 1 participant had chronic HCV infection but had not been treated, 1 had tested HCV antibody positive and did not have active infection, and 2 others who had chronic HCV infection had been successfully treated and cleared the virus.

### Individual barriers to HCV testing and treatment

#### Limited knowledge and misconceptions regarding HCV

At the individual level of analysis, limited knowledge of the main modes of HCV transmission (e.g., sharing needles, blood transfusion), HCV diagnosis, and the availability of treatment for HCV were barriers. Some participants believed that HCV is transmitted through fecal matter, such as from exposure to fecal matter on toilet seats and chairs, and casual airborne contact with residents at shelters. There was a general fear of exposure to infectious diseases other than HCV, in particular hepatitis A virus (HAV) and hepatitis B virus (HBV) infection, while living in shelters. Participants also expressed confusion about the differences between HAV, HBV, and HCV infection and had misconceptions about how these viruses were transmitted and the health consequences of infection

*“(HCV) It can be transmitted through fecal matter. You have to be really careful of your environment.” (White Woman).*

*[HCV can also be transmitted] “if someone doesn’t wash their hands in a restaurant.” (African American Woman).*

Many participants volunteered that they had been tested with rapid HCV tests or through traditional blood draws in the past, but some participants expressed misunderstanding of the meaning of a positive HCV antibody test result. Others did not know which tests were used to diagnose HCV infection or understand that HCV diagnosis involved a two-step process in which antibody testing is followed by HCV ribonucleic acid (RNA) testing for confirmation of active infection in antibody positive patients. There was also confusion between receipt of HCV testing in the past and instances in which participants had received HAV and HBV vaccination. Additionally, participants also did not understand that individuals can clear HCV or have an active/chronic infection that continues to adversely affect their liver and overall health. Finally, some participants were not aware about advances in HCV treatment, and the availability of new pharmacotherapies that cure HCV in most patient populations [24].

*“Is it a blood test only, saliva or swab?” (Hispanic Man).*

*“You can be a carrier … .and you can have it (HCV). It can lay dormant in your system for a while.” (White Woman).*

*“Is there a cure or treatment?” (Hispanic Man).*

Participants raised concerns about their risk of contracting HCV infection and about behaviors that may increase their risk for infection. Some participants reported seeking testing for HCV because they had engaged in risk behaviors in the past, including engaging in unprotected sex and injection drug use, while others reported that they did not feel the need to get tested because they did not perceive they were at risk. Some participants reported that some people avoided HCV testing because they feared receiving a positive test result and the consequences of being infected.

*“I got tested because I had a blood transfusion.” (African American Woman).*

*“They (homeless client) don’t want to know … because it could really change someone, and in a good or a bad way. They are afraid of dying.” (White Man).*

#### Mistrust of health care providers

Mistrust of health care providers and government institutions was a barrier for treatment. A male participant questioned whether transmission of HCV through blood transfusions had been an intentional act to harm people. A female participant expressed skepticism about messaging or advice given by the medical community regarding HCV. She described that receiving inconsistent information about how she became infected with HCV and new information about available treatments contributed to uncertainty about whether to trust medical advice. The mistrust of providers was also reported as a reason why people do not seek treatment for HCV.

*“My view of most doctors … , they are mostly pharmaceutical sales reps instead of doctors. They will write you a script...” “That needs to be taken care of because that is a trust issue right there and a very serious one.” (African American Man).*

*“They’re making you a guinea pig. I don’t trust [them]. Because this trust has been breached already” (African American Man).*

#### Active substance use, mental illness, and chronic health conditions

Active drug use was referenced as a barrier to HCV testing. Participants discussed that those with drug dependency may not be ready to address HCV because they were focused on managing drug withdrawal symptoms and controlling their drug use. In addition, participants viewed mental illness and having psychotic symptoms as an important barrier to HCV testing and treatment. They expressed the belief that individuals with mental illness were not concerned about their physical health status because they were not able to make informed decisions about their health. Some participants also expressed the belief that those who had another serious chronic medical condition, such as HIV infection, which was perceived to be a more serious and stigmatized condition than other chronic medical conditions, would be less concerned about knowing their HCV status.

*“Some people are stuck in addiction, that they’re not even caring about it [other medical conditions], they’re just looking towards the next fix or their next high (White Man).”*

*“Mental health issues. There are some people with [mental illness], they don’t care that they have any disease or not. They don’t care about their surroundings (African American Woman).”*

### System barrier to HCV testing and treatment

#### Limited advocacy for HCV services by shelter staff

At a system level of analysis, some participants reported the sentiment that there was limited advocacy for HCV services by shelter staff. Although the shelter provided onsite HCV testing in collaboration with a community-based social service agency, participants believed that the expansion of HCV services, including treatment in the shelter was not a high priority for the shelter staff. Shelter policies and rules, including curfews were described as reasons why clients were asked to leave the shelter, and acknowledged as factors that could contribute to a person’s ability to complete a course of HCV treatment in the shelter setting.

“*You will hear the word no so many times in one day*. *(African American Man*)”.

### Societal barrier to HCV testing and treatment

#### Social stigma against homeless individuals

The discussion of stigma centered on participants’ views that society and the media hold a negative view of people who are homeless. They described that being homeless was stigmatizing and affected many areas of their lives, including their ability to get treatment for HCV.

*“[The homeless is] A community that continues not to get the attention that it really needs. …*. Y*ou tell somebody you’re in the shelter, that’s like a disease now. They turn their backs on you … The way the media puts out information about the homeless, it’s like negative press … That word alone serves like a sentence.” (African American Man).*

### Individual facilitator to HCV testing and treatment

#### Motivators for HCV testing and treatment

Participants acknowledged the benefits of both HCV testing and treatment. They reported that a strong personal motivator to be tested and treated for HCV was to prevent transmission to others and prevent disease progression. They reported that HCV education as a key factor that may motivate some people to be tested and treated for HCV. In particular, they expressed the high importance of disseminating information that a cure for HCV exists. Furthermore, some suggested that knowing the adverse health consequences of untreated HCV might motivate some to seek treatment.

*“What motivated me to get tested is so it doesn’t spread.” (White Man).*

“*… I want to be around for my family. I want them to come around me and I don’t want to expose them.”(African American Woman).*

*“They need to know there is a cure.”(Hispanic Man).*

### System facilitator to HCV testing and treatment

#### Financial incentives

Financial incentives was cited as a strong motivator to engage homeless clients in HCV rapid testing and receipt of HCV education. Participants reported that $10 gift cards offered by community–based outreach HCV testing programs motivated some to get tested with the rapid HCV test.

*“People will get it [testing] as long as there is an incentive behind it so if there’s no incentive they won’t get it.”(African American Woman).*

### Societal facilitators for HCV testing and treatment

#### Prior experience with rapid HCV testing

Participants had learned about HCV infection through a variety of sources including from the media, family members who were infected with HCV, mobile testing vans, federal prison, community health centers and the Veterans Health Administration, college coursework, and through HCV testing initiatives in the homeless shelter. Many had been tested for HCV in the past with the rapid HCV antibody test. Participants reported familiarity with community-based staff and rapid testing procedures encouraged future testing. Those who received rapid HCV antibody testing from community-based outreach programs commonly reported receiving financial incentives for HCV testing. Others received HCV testing based on health care provider recommendations due to the presence of risk factors.

*“Community testing programs offers [HCV testing] sometimes and nowadays you can, if, you are worried about it, … or you think you have been with someone who might have it, you can go to the community clinic and they will give you the test.” (White Woman).*

*Availability of affordable direct acting antiviral treatment.*

Most participants were knowledgeable about public assistance programs and resources available to pay for DAA medications. Participants’ ability to pay for treatment and the cost of DAAs were not considered significant barriers to HCV treatment; they believed that treatment for HCV infection with DAAs was free if someone decided to seek treatment.

*“The first thing that came through my mind was how was I going to afford this medication? Does Medicaid cover [the medication]?...If you don’t know what to ask for [the doctor], a lot of people will turn around and walk out the door.” (White Woman).*

*“Cost has nothing to do with it. It’s usually free. It’s not about cost; it’s trust.” (African American Man).*

## Discussion

We sought to identify barriers and facilitators to HCV testing and treatment among homeless shelter clients at the individual, system, and societal levels. Our study builds on a prior study conducted before the availability of once-daily DAA treatment of a sample (N = 240) of homeless HCV-infected patients receiving care at a large FQHC [25]. In that study, despite the multiple barriers associated with homelessness and substance use, Beiser et al. found that the 86.5% of the sample expressed an interest in receiving HCV treatment, and among high-risk subgroups including respondents living with HIV and those reporting illicit drug use in the past 30 days over two-thirds reported an interest in receiving treatment, 66.7 and 65.9%, respectively. In our analysis, individual- and social-level factors were predominant barriers affecting homeless persons’ decisions to engage in HCV prevention and treatment. Specifically, individual-level factors commonly reported by this sample of homeless adults were limited HCV knowledge and misconceptions about HCV infection, medical mistrust, as well as co-occurring substance use, psychiatric and chronic medical conditions. In addition, social level factors of stigma and discrimination that have been linked to low uptake of HCV testing and treatment among other marginalized populations, such as injection drug users [26], were prevalent among these participants. Notably, although a lack of health insurance is a well-documented system-level barrier to HCV care [14, 27], participants did not perceive the cost of DAA treatment, nor strict requirements from insurance companies or Medicaid programs for DAA treatment coverage, as barriers to care [28].

Although the study of HCV-infected homeless adults receiving care at a large FQHC conducted by Beiser et al. found that almost half (45.7%) of the respondents had good knowledge of HCV [25], participants in this study had limited knowledge specifically related to modes of transmission, interpretation of HCV screening tests, and disease consequences. Our findings highlight that knowledge of HCV among homeless adults remains low, and supports prior studies conducted in the interferon-based treatment era that have shown significant gaps in knowledge and misconceptions about HCV risk factors, prevention, transmission and treatment among homeless, and other at risk populations [29, 30].This lack of in-depth knowledge of HCV may contribute to low rates of DAA treatment, continued transmission, and worse health outcomes. Having greater knowledge of HCV is associated with an increased willingness to undergo HCV treatment [31]. Our results speak to the critical need to engage at risk homeless adults in HCV educational programs embedded in settings that they frequently access, such as homeless shelters, as this population tends to lack regular access to healthcare where individuals would traditionally receive HCV prevention education.

Our data show some support for the provision of HCV care within homeless shelters. Participants reported that financial incentives may prove particularly useful in promoting uptake of HCV rapid testing and education in the shelter setting. Financial incentives may serve as a useful tool for engaging at risk people in HCV testing [32], such as homeless adults. As many participants had prior experience with HCV rapid testing, scaling up HCV prevention services so that they become a routine part of the intake process could be one way to ensure that those at highest risk of infection and transmission are tested and linked to care. However, interventions to address HCV in shelter settings must consider prior stigmatizing or negative experiences with health care providers and systems. Stigma of homelessness may negatively impact health seeking behavior, and experienced stigma in and of itself represents an added burden above and beyond the challenges associated with the experience of homelessness [33]. Although we do not have information about participants’ stigmatizing experiences with health care providers, mistrust of providers discussed in focus groups may reflect prior negative experiences with providers in health care settings. Our findings highlight the need to address stigma in this vulnerable population through culturally informed treatment that is based on a trusting relationship between the patient, provider, and health care system [34].

This study reflects the views of homeless clients recruited from a large shelter in San Francisco, thus findings may not be applicable to other geographic locations of the country. Specifically, in states that impose strict DAA treatment eligibility criteria for Medicaid coverage, barriers related to the cost of treatment and insurance coverage may become more prominent for homeless adults [28]. Since we assessed perceptions regarding both HCV testing and care, participation in focus groups was not limited to people diagnosed with chronic HCV infection. Thus, we are not able to provide a more in-depth exploration of homeless individuals living with HCV and their experiences with engagement in DAA treatment. Future studies should seek to provide further insight about the challenges of people who are homeless in accessing HCV care in other urban and rural geographic regions of the country. Despite these limitations, the exploration of perspectives among homeless persons at risk for HCV infection regarding barriers to HCV care can be instrumental in informing the design of tailored interventions to improve access to HCV prevention and treatment services in non-traditional health care settings.

## Conclusions

In conclusion, we identified several factors that impede HCV screening and treatment among homeless adults and which must be addressed in designing models of HCV care for homeless populations. The integration of HCV care within homeless shelters that includes HCV education, point-of-care testing, and free evaluation and treatment may be an effective way of facilitating access to life-saving care for a vulnerable population of HCV infected persons.

## Supplementary information

**Additional file 1.**

## Data Availability

The datasets generated during and/or analyzed during the current study are not publicly available due their highly sensitive nature. Reasonable requests for access will be considered by the principal investigator.

## Abbreviations

HCV: hepatitis C virus
DAA: direct acting antiviral
FQHC: federally qualified health centers
HBF: Health Behavior Framework
HAV: hepatitis A virus
HBV: hepatitis B virus
RNA: ribonucleic acid
