# Supplementary material for: Clients’ perceptions of barriers and facilitators to implementing hepatitis C virus care in homeless shelters
Source: BMC Infect Dis. 2020 May 29;20:386. doi: 10.1186/s12879-020-05103-6 (PMC7260732; doi:10.1186/s12879-020-05103-6)
Supplement: Supplementary file 1 — Additional file 1. [file 12879_2020_5103_MOESM1_ESM.docx]

Focus group instrument (abridged)

1. In general, what are some reasons you have for taking care of your health?
2. Where are you getting medical care?
3. Are you happy with your experiences? Why or why not?
4. Have you heard of hepatitis C?
5. What do you think causes hepatitis C?
6. Are you worried about getting hepatitis C?
7. What do you think are some reasons that people experiencing homelessness have for getting a hepatitis C test?
8. What do you think are some reasons that people experiencing homelessness or people with unstable housing have for NOT getting a hepatitis C test?
9. What do you think are some reasons that people experiencing homelessness or people with unstable housing with hepatitis C have for getting hepatitis C treatment?
10. What do you think are some reasons that people experiencing homelessness or people with unstable housing have for NOT getting hepatitis C treatment?
11. What kind of help do you think that people who are homeless need to successfully complete hepatitis C treatment?
